# Early and Long-Lasting Hematologic Recovery in a Young Adult With Severe Aplastic Anemia Treated With Romiplostim, Horse-Anti-Thymocyte Globulin (ATG), and Cyclosporine A: A Case Report

**DOI:** 10.7759/cureus.94634

**Published:** 2025-10-15

**Authors:** Eichi Kakizaki, Takehiro Higashi, Kenichi Akao, Hiroaki Morimoto, Junichi Tsukada

**Affiliations:** 1 Department of Hematology, University of Occupational and Environmental Health, Japan, Kitakyushu, JPN

**Keywords:** aplastic anemia, bone marrow failure, cyclosporine a, first-line therapy, hematologic recovery, horse atg, immunosuppressive therapy, romiplostim, severe pancytopenia, young adult

## Abstract

Aplastic anemia (AA) is a rare but potentially life-threatening bone marrow failure syndrome, typically characterized by peripheral pancytopenia and marked marrow hypocellularity. In acquired cases, immune-mediated destruction of hematopoietic stem cells is the predominant mechanism; however, intrinsic stem cell defects may also contribute to marrow failure, particularly in inherited bone marrow failure syndromes. Other etiologies, such as drug-induced marrow suppression, viral infections, and radiation exposure, should also be considered in the differential diagnosis.

We present the case of a 22-year-old male with newly diagnosed severe AA, who exhibited pancytopenia and hypocellular marrow devoid of dysplastic features or blast cells. Cytogenetic studies and viral serologies yielded unremarkable results. Inherited marrow failure syndromes were excluded based on clinical phenotype, cytogenetic analysis, and absence of family history, supporting the diagnosis of acquired AA.

Given the lack of an HLA-matched sibling donor, combination therapy consisting of horse anti-thymocyte globulin (hATG), cyclosporine A (CsA), and romiplostim (ROMI) was promptly initiated. Hematologic improvement was observed by week two, and complete response, defined as hemoglobin ≥100 g/L, absolute neutrophil count ≥1.0 ×10⁹/L, and platelet count ≥100 ×10⁹/L-was achieved by week five. Transfusion independence was attained early in the course, and no serious adverse events were observed.

This case underscores the potential utility of early triple immunosuppressive therapy (IST) in treatment-naïve severe AA, particularly among younger patients, where prompt immunosuppressive intervention may accelerate hematologic recovery and support the preservation of marrow architecture.

## Introduction

Acquired aplastic anemia (AA) is a rare but potentially life-threatening hematologic disorder, characterized by bone marrow failure resulting in peripheral pancytopenia and profound marrow hypocellularity. Although its etiologies are diverse, substantial evidence implicates immune-mediated mechanisms in the majority of acquired cases, particularly cytotoxic T-cell-mediated destruction of hematopoietic progenitors and the aberrant overproduction of inhibitory cytokines, including interferon-γ (IFN-γ) and tumor necrosis factor-α (TNF-α) [1]. However, not all cases are immune-mediated; other potential etiologies include drug-induced marrow suppression (e.g., chloramphenicol, anticonvulsants), chemical toxins (e.g., benzene), viral infections (e.g., Epstein-Barr virus, human immunodeficiency virus, hepatitis B virus, and hepatitis C virus), radiation exposure, and inherited bone marrow failure syndromes should also be considered in the differential diagnosis [1].

Clinically, AA may present with nonspecific manifestations such as fatigue or bleeding tendencies; however, it can rapidly progress to severe cytopenia that carries a substantial risk of life-threatening infections, hemorrhagic complications, and transfusion dependence. Thus, timely diagnosis and prompt initiation of treatment are critical to improving patient outcomes. Standard first-line therapy includes anti-thymocyte globulin (ATG) combined with cyclosporine A (CsA), or hematopoietic stem cell transplantation (HSCT) in patients with an available HLA-matched sibling donor.

Thrombopoietin (TPO), primarily synthesized by hepatic cells, regulates platelet production through the activation of its receptor c-MPL, which is expressed not only on the megakaryocyte lineage but also on hematopoietic stem and progenitor cells (HSPCs) [2]. Accordingly, TPO signaling plays an integral role in stem cell proliferation and multilineage hematopoiesis.

Romiplostim (ROMI), a fusion protein consisting of an Fc fragment linked to a TPO receptor-binding peptide, specifically targets the extracellular domain of c-MPL. It was approved for use in refractory AA in Japan in 2019 and later for treatment-naïve cases in 2022. In a global phase II/III clinical trial involving patients with immunosuppressive therapy (IST)-refractory AA, ROMI demonstrated a hematologic response rate of 83.9% at Week 27 [3].

Prior to July 2023, rabbit-derived ATG (rATG) was the only form of ATG available in Japan for AA treatment. The approval of horse-derived ATG (hATG) in July 2023, alongside ROMI’s expanded indication, has facilitated a broader range of therapeutic strategies.

One such strategy includes the combination of hATG, CsA, and ROMI, which was adopted in the present case. Although ROMI has recently emerged as a promising adjunct to IST in severe AA, its use has been primarily reported in pediatric populations and in combination with hATG, where favorable hematologic responses have been observed [4].

However, its upfront application in adult patients, particularly in combination with hATG, remains largely undocumented. This lack of prior evidence highlights the novelty of our case, which demonstrates the feasibility and potential efficacy of this triple regimen in adult-onset severe AA. By bridging this gap, our report contributes new insights into the therapeutic landscape for adult patients with newly diagnosed severe AA. To our knowledge, no prior reports have described the use of ROMI in combination with hATG and CsA as first-line therapy in adult severe AA. This case illustrates an early and sustained hematologic recovery following this novel therapeutic approach.

Herein, we present a case of newly diagnosed severe AA in a young adult who received first-line IST comprising hATG, CsA, and ROMI, resulting in rapid hematologic recovery and early transfusion independence. Although the patient met the Japanese criteria for Stage 3 (moderately severe) AA, this category is often considered part of the “severe AA spectrum” in clinical practice due to transfusion dependence and multilineage cytopenia. These clinical findings suggest that early implementation of triple IST may offer significant therapeutic advantages, particularly in cases where HSCT is not feasible.

## Case presentation

A 22-year-old male presented with exertional palpitations persisting over several weeks. On admission, he was alert and hemodynamically stable, exhibiting mucocutaneous pallor and petechiae. Notably, no fever, lymphadenopathy, or hepatosplenomegaly was observed. The patient denied any history of exposure to offending drugs (e.g., chloramphenicol, anticonvulsants), chemical toxins (e.g., benzene), or radiation. Routine urinalysis and renal function tests were unremarkable, and viral serologies were non-reactive. Initial laboratory assessment revealed pancytopenia, including an absolute neutrophil count of 0.66 × 10⁹/L, consistent with increased susceptibility to infection, with pertinent data summarized in Table 1.

**Table 1:** Hematologic Parameters at Diagnosis. Abnormal values are annotated with “H” (high) or “L” (low) relative to the reference range.

Blood Parameters	Value	Normal Range
White blood cell count (×10^9^/L)	2.2 L	3.3-8.6
Neutrophils (%)	30 L	38.0-74.0
Lymphocytes (%)	69 H	16.5-49.5
Monocytes (%)	1 L	2.0-10.0
Eosinophils (%)	1	0-8.5
Basophils (%)	1	0-2.5
Red blood cell count (×10^12^/L)	1.37 L	4.35-5.55
Hemoglobin (g/dL)	5.3 L	13.7-16.8
Hematocrit (%)	15.6 L	40.7-50.1
Reticulocytes (×10^9^/L)	18 L	20-100
Platelet count(×10^9^/L)	31 L	158-348
Prothrombin time (sec)	11.6	11.0-13.4
International normalized ratio	0.97	0.94-1.15
Partial-thromboplastin time (sec)	33.2	24.0-39.0
Fibrinogen (mg/dL)	206	200-400
Fibrin degradation product (μg/dL)	<3.0	<5.0
Total protein (g/dL)	6.6	6.6-8.1
Albumin (g/dL)	4.9	4.1-5.1
Urea nitrogen (mg/dL)	17	8.0-20
Creatinine (mg/dL)	0.85	0.65-1.07
Uric acid (mg/dL)	3.4	3.7-7.8
Total bilirubin (mg/dL)	0.8	0.4-1.5
Aspartate aminotransferase (U/L)	16	13-30
Alanine aminotransferase (U/L)	23	10-42
Lactate dehydrogenase (U/L)	132	124-222
Alkaline phosphatase (U/L)	65	38-113
C-reactive protein (mg/dL)	0.03	≦0.14
Sodium (mmol/L)	139	138-145
Potassium (mmol/L)	3.9	3.6-4.8
Chloride (mmol/L)	104	101-108
Calcium (mg/dL)	9.1	8.8-10.1
Iron (μg/dL)	251 H	40-188
Ferritin (ng/mL)	591 H	21-282
Vitamin B12 (pg/mL)	163 L	233-914
Folate (ng/mL)	4.8	3.6-12.9

**Figure 1:**
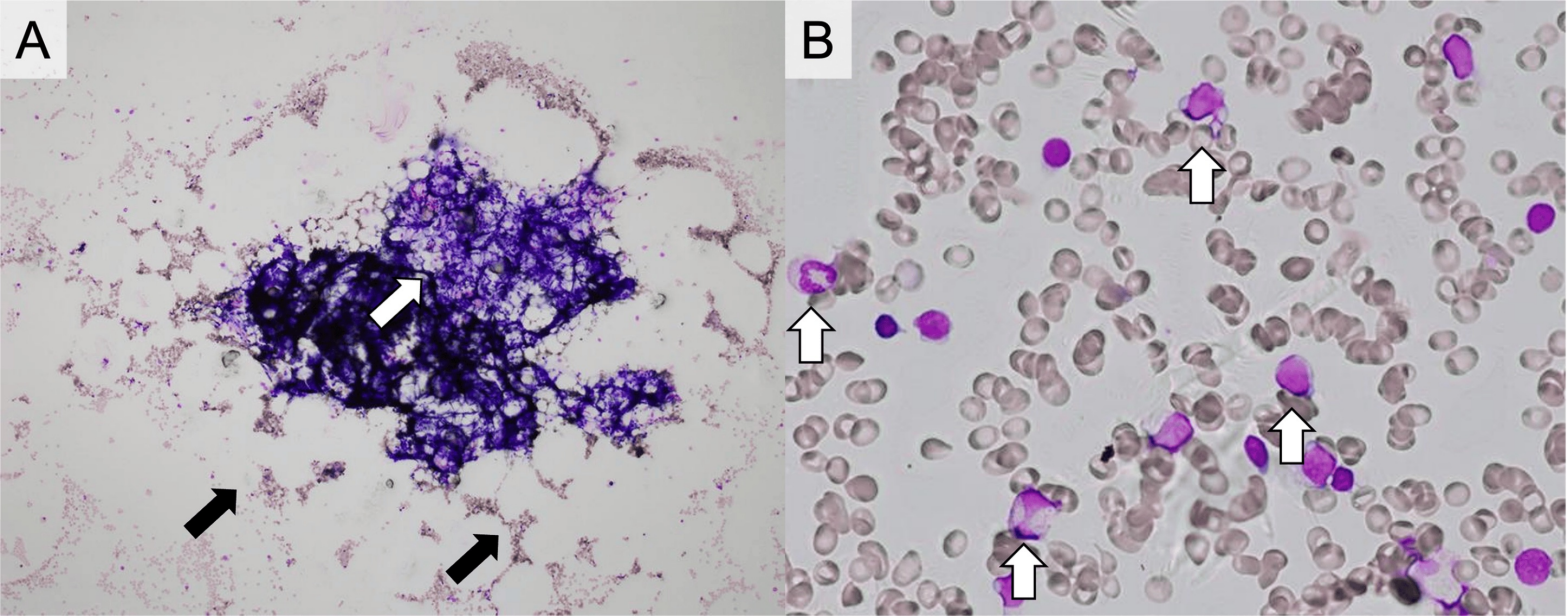
Bone Marrow Aspirate Findings at Diagnosis (A) Marked hypocellularity with extensive fatty marrow replacement. White arrows indicate residual stromal components, while black arrows highlight areas of fatty replacement consistent with marrow hypocellularity. (B) No evidence of dysplasia, blasts, or clonal abnormalities.

Bone marrow aspiration demonstrated marked hypocellularity accompanied by fatty replacement, with no evidence of blast proliferation or dysplastic changes (Figure 1).

**Figure 2:**
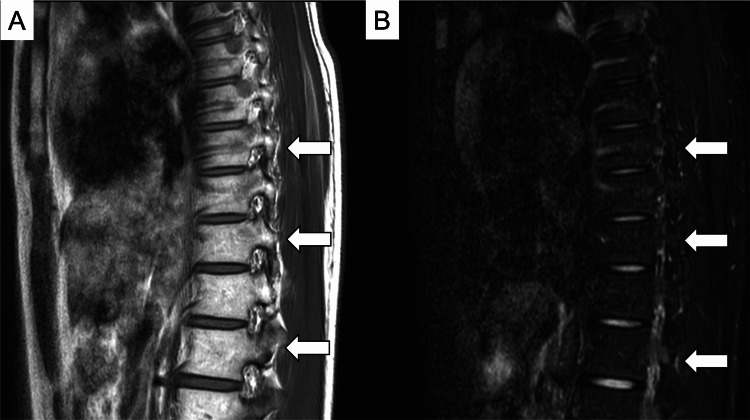
Magnetic Resonance Imaging (MRI) of the Thoracic Spine T1-weighted (A) and short-tau inversion recovery (STIR) images (B) demonstrating high T1 and low STIR signal intensity, indicative of fatty marrow infiltration compatible with aplastic marrow phenotype.

Cytogenetic analysis revealed a normal karyotype, and viral serologies were non-reactive, excluding infectious etiologies. The presence of paroxysmal nocturnal hemoglobinuria (PNH)-type cells, as detected by flow cytometry, is frequently observed in patients with immune-mediated AA and supports the diagnosis of acquired AA. These cells reflect a clonal population deficient in GPI-anchored proteins, which may arise due to immune escape mechanisms in the context of marrow failure. The diagnosis of moderately severe AA (Stage 3) was established in accordance with the Japanese severity classification criteria, which define this stage as meeting at least two of the following: reticulocyte count <60 × 10⁹/L, absolute neutrophil count <1.0 × 10⁹/L, and platelet count <50 × 10⁹/L, with transfusion dependence of ≥2 units of red blood cells per month. Although classified as Stage 3, this category is often considered part of the “severe AA spectrum” in clinical practice due to transfusion dependence and multilineage cytopenia. Furthermore, magnetic resonance imaging (MRI) of the thoracic spine, including T1- and short-tau inversion recovery (STIR)-weighted sequences, showed high T1 signal intensity and corresponding low STIR signal intensity, findings consistent with fatty marrow replacement (Figure 2).

**Figure 3:**
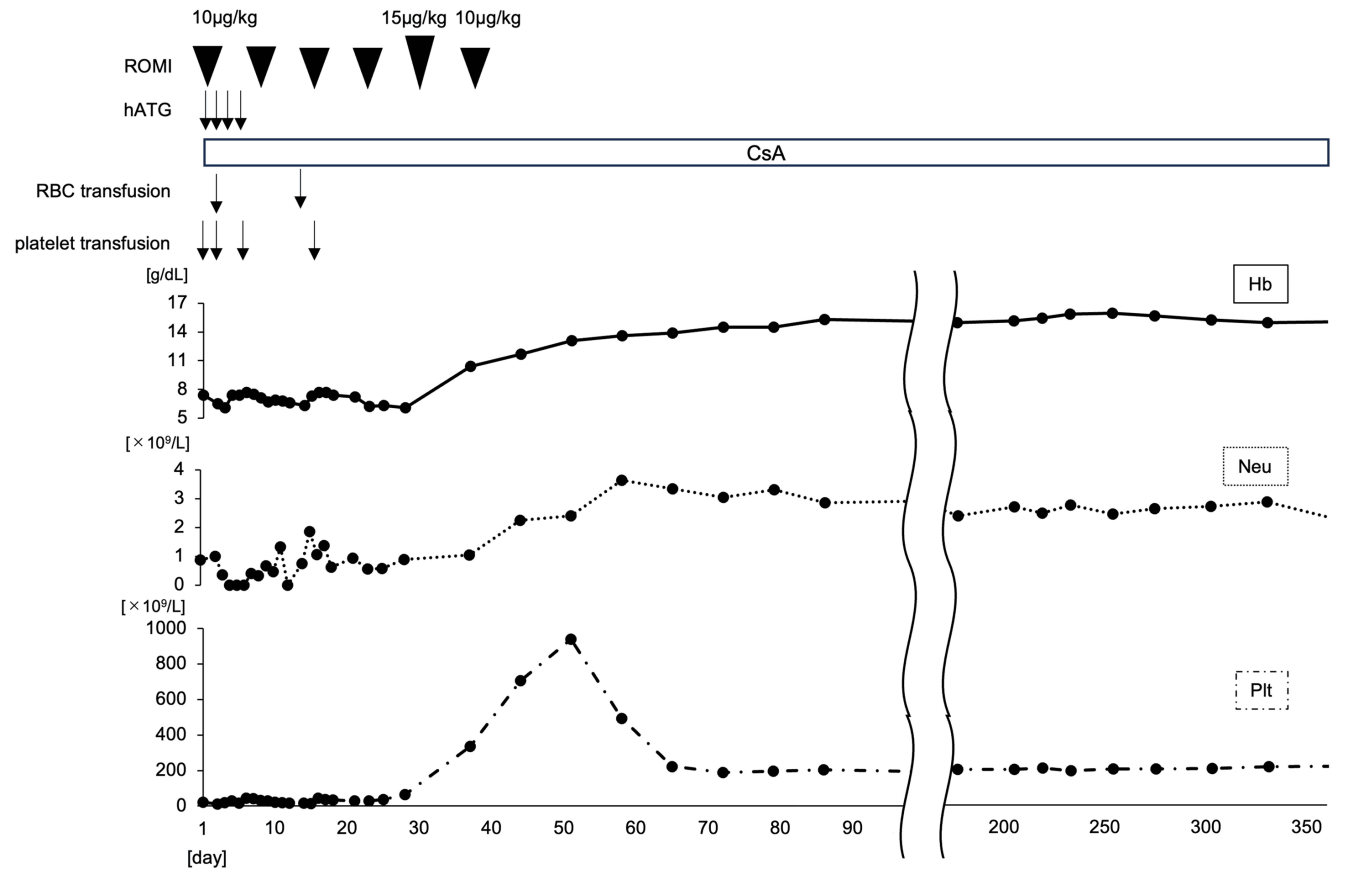
Hematologic Response Following Triple Immunosuppressive Therapy (IST) Time course of hematologic recovery following treatment with Horse anti-thymocyte globulin (hATG), Cyclosporine A (CsA), and romiplostim (ROMI). Trends in hemoglobin, platelet count, and absolute neutrophil count reflect complete hematologic recovery.

In the absence of an HLA-matched sibling donor, the patient was initiated on first-line IST comprising a triple-agent regimen that included hATG at 40 mg/kg/day administered intravenously over four consecutive days, CsA given orally with dose adjustments to maintain a two-hour post-dose concentration of at least 600 ng/mL, and ROMI administered as weekly subcutaneous injections at a dose of 10-15 μg/kg. Triple IST was initiated on Day 1 and resulted in early hematologic improvement (Figure 3).

By week 2, peripheral blood counts began to recover, with hemoglobin at 77 g/L, white blood cell (WBC) count at 2.2 × 10⁹/L, and platelet count at 45 × 10⁹/L (Table 2). By week 5, the patient achieved a complete response, defined by hemoglobin of 104 g/L, WBC of 3.2 × 10⁹/L, and platelet count of 335 × 10⁹/L, meeting established international response criteria (Table 2).

**Table 2:** Longitudinal Comparison of Hematologic Parameters During the Triple Immunosuppressive Therapy (IST).

	Normal Range	On Admission	2 Weeks after the Initiation of the IST	2 Months after the Initiation of the IST	6 Months after the Initiation of the IST	1 Year after the Initiation of the IST
Hemoglobin (g/dL)	13.7-16.8	5.3	7.7	11.7	14.9	15
Platelet count (×10^9^/L)	158-348	34	45	707	202	219
Leukocytes (×10^9^/L)	3.3-8.6	2.2	2.2	7.7	3.5	3.6
Neutrophils (×10^9^/L)		0.73	1.05	5.39	2.32	2.20

Transfusion independence was achieved early in the treatment course, accompanied by marked improvement in bleeding tendency. Although the patient did not present with active infection at diagnosis, the profound neutropenia (absolute neutrophil count 0.66 × 10⁹/L) carried a substantial theoretical risk of infectious complications. The observed recovery in neutrophil counts following IST likely contributed to a reduction in infection risk, even in the absence of documented infectious episodes. In addition to the mild febrile episode and rash observed in this case, other adverse drug reactions (ADRs) associated with triple IST may include serum sickness, anaphylaxis, nephrotoxicity, hypertension, and clonal evolution. While none of these events occurred in the present case, close monitoring remains essential, particularly during the early treatment phase.

At week 5, the platelet count exceeded 200 × 10⁹/L, prompting a dose reduction of romiplostim in accordance with the prescribing information. Weekly platelet monitoring was implemented, and romiplostim was subsequently discontinued as platelet counts continued to increase beyond target levels. Importantly, no thrombotic complications occurred during the period of elevated platelet levels. A proactive safety strategy - including regular clinical assessments and laboratory monitoring - was employed to ensure patient safety (Figure 3). The patient was discharged on Day 46 following confirmation of transfusion independence and hematologic stability. Outpatient follow-up was conducted weekly for the first two months, biweekly for the next month, and monthly thereafter. At the latest follow-up, performed 20 months after treatment initiation, the patient remained in complete hematologic remission without recurrent cytopenia or clonal evolution, with no evidence of bone marrow failure and sustained transfusion independence under ongoing CsA therapy.

## Discussion

In patients with severe AA who lack a suitable donor, IST remains the mainstay of initial treatment. Since 2014, accumulating evidence has demonstrated the efficacy of eltrombopag (EPAG) - a TPO receptor agonist (TPO-RA) - in both treatment-naïve and refractory AA populations [5-9]. Notably, Olnes et al. reported that EPAG monotherapy facilitated trilineage hematopoietic recovery and improved bone marrow cellularity [6]. In a more recent study, Peffault de Latour et al. showed that adding EPAG to IST significantly increased the complete response (CR) rate at three months compared to IST alone (22% vs. 10%) [7].

ROMI, another TPO-RA, has demonstrated promising efficacy, particularly in IST-refractory AA, and its role in upfront therapy is now being explored [4, 10-13]. A multicenter phase II/III trial reported an overall hematologic response rate of 83.9% in IST-refractory patients treated with ROMI [12]. Additionally, ROMI administered in combination with CsA at doses ranging from 10 to 20 μg/kg achieved a 27-week response rate of 41.7% in IST-naïve patients, with most adverse events graded as mild [13].

Sharathkumar et al. further described four pediatric cases receiving triple IST (hATG, CsA, and ROMI), of which two attained CR, and no serious adverse events occurred during the course of treatment [4]. Although the patient met the criteria for Stage 3 AA under the Japanese severity classification, the transfusion dependence and profound cytopenia warranted prompt initiation of triple IST, aligning with clinical practices for severe AA. The patient described in the present report demonstrated a favorable hematologic response to the triple IST, which was well tolerated without major complications. Reports demonstrating the efficacy of this combination therapy in adult patients with severe AA are extremely limited, and this case may represent one of the first documented examples.

Prompt initiation of IST appears to be a key determinant of clinical response and long-term outcome. In this case, hATG, CsA, and ROMI were administered shortly after diagnosis, resulting in CR by week five. This finding aligns with prior observations, including those by Peffault de Latour et al., indicating that delays in initiating IST beyond three months may be associated with inferior prognosis and reduced response rates [7]. Recent data from Jang et al. identified important predictive factors for ROMI responsiveness, including shorter duration from diagnosis, elevated baseline reticulocyte counts, and higher platelet counts [14]. These findings further support the clinical rationale for early therapeutic intervention in AA, underscoring the importance of timely IST initiation to optimize hematologic outcomes. Furthermore, the choice of hATG over rATG was supported by randomized data from Scheinberg et al., demonstrating superior hematologic response and overall survival with hATG in treatment-naïve patients with severe AA [15]. These findings reinforce the importance of selecting the appropriate immunosuppressive regimen early in the disease course. Additionally, flow cytometric detection of PNH-type cells in this case may further support the diagnosis of immune-mediated marrow failure. As described by Young, AA and PNH share a close pathophysiologic relationship, with small PNH clones frequently observed at diagnosis [1]. Their presence may reflect immune escape mechanisms and has been associated with a favorable response to immunosuppressive therapy.

First-line IST induces hematologic recovery in approximately 60% of AA patients [16, 17], with long-term survival outcomes varying by age group: 82% in individuals aged 1-20 years, 69% in those 21-40 years, and 58% in patients over 40. Medinger et al. proposed that enhanced bone marrow microenvironment integrity in younger patients may contribute to more favorable responses [18], which likely played a role in the outcome observed in this 22-year-old case.

Despite their therapeutic benefits, TPO-RAs carry a potential risk of clonal evolution toward Myelodysplastic Syndromes (MDS) or Acute Myeloid Leukemia (AML). Winkler et al. reported that 19% of IST-refractory patients treated with EPAG developed cytogenetic abnormalities, including five cases of monosomy 7, typically arising within six months [19]. They hypothesized that these aberrations might reflect clonal expansion rather than de novo mutation.

Next-generation sequencing studies have revealed that clonal hematopoiesis is present in over 70% of AA cases, frequently involving mutations in ASXL1, BCOR, and BCORL1. Although many of these clones remain clinically indolent, their prevalence illustrates the underlying immune-mediated pathophysiology of AA. Babushok demonstrated that younger patients tend to carry fewer high-risk mutations associated with myeloid transformation-22% in those under 20 years compared to 39% in adults [20].

With respect to ROMI, cytogenetic abnormalities appear less common. Jang et al. reported monosomy seven in only one of 31 IST-refractory patients treated with ROMI [3]. Given the direct stimulatory effects of TPO-RAs on HSPCs, continuous molecular surveillance remains imperative.

Collectively, these observations suggest that the favorable outcome in this case may be attributed to the patient's young age, favoring a regenerative marrow microenvironment, and timely administration of triple IST. These factors likely contributed to rapid hematologic recovery while limiting clonal expansion. The present findings support the notion that early use of hATG, CsA, and ROMI may optimize therapeutic efficacy and mitigate the risk of clonal progression, particularly in younger individuals. Incorporating molecular monitoring alongside timely immunosuppressive intervention may offer a personalized and proactive approach to managing severe AA. To our knowledge, this may represent one of the first documented adult cases of severe AA successfully treated with upfront triple IST incorporating ROMI, highlighting its potential as a first-line option.

## Conclusions

This case underscores the therapeutic potential of integrating TPO-RAs into first-line IST, especially for younger patients with severe AA lacking a matched donor. Early initiation of hATG, CsA, and romiplostim yielded rapid hematologic improvement and transfusion independence without serious adverse events. While further prospective studies are warranted to refine the optimal timing, dosing strategies, and patient selection criteria, early triple IST may not only enhance short-term hematologic outcomes but also inform future therapeutic strategies, particularly for younger patients with limited donor availability.
